# Simultaneous Bilateral Ovarian Torsion in a Transgender Patient

**DOI:** 10.7759/cureus.28972

**Published:** 2022-09-09

**Authors:** Rasheeda Rickman, Alanna O'Connell, Meredith Jones, John Morrison

**Affiliations:** 1 Emergency Medicine, Brookdale University Hospital Medical Center, Brooklyn, USA

**Keywords:** female to male transgender, obgyn, bilateral ovarian torsion, emergency medicine, transgender medicine

## Abstract

Ovarian torsions are a diagnostic challenge in the emergency room that result in significant morbidity when missed. Ovarian torsions have a low incidence and can present with atypical and variable symptoms. As such, recognition of ovarian torsion is a key skill that emergency clinicians must master.

Typically, ovarian torsion is a unilateral finding but there have been cases of bilateral torsions noted previously in the literature. These rare cases tend to have higher morbidity making it even more important to recognize. This case is unique in that it was a transgendered patient developing synchronous bilateral ovarian torsions, which, to our knowledge, has not been previously described in the literature. This case illustrates the potential for unique presentations of this condition. We advise a high index of suspicion until more data, both objective and subjective gestalt, is known.

## Introduction

Ovarian torsions are a diagnostic challenge in the emergency department that result in significant morbidity to the patient when missed. Ovarian torsions have a low incidence and can present with atypical and variable symptoms [1]. It represents a disability risk in terms of fertility. It also presents a significant risk of mortality, especially if the underlying etiology is an ectopic pregnancy, as progressive ischemia leads to rupture and internal hemorrhage. Additional mortality risk can be considered longitudinally in terms of estrogen’s effect on cardiovascular and bone health. As such, recognition of ovarian torsion is a key skill that emergency clinicians must master. Fortunately, these patients tend to trigger a physician’s gestalt as they usually present with notable patient distress, nausea and vomiting, and classic history of sudden-onset pain.

Overwhelmingly, ovarian torsion is a unilateral finding. However, bilateral ovarian torsion is not unknown. These cases, while rare, cause significantly more morbidity. Previous cases of bilateral torsion have been reported in pediatrics [2,3], in cases of patients with polycystic ovarian syndrome (PCOS) [4], or with asynchronous presentations [2,3,5,6]. There are no reports of transgendered patients developing ovarian torsions, much less so simultaneous bilateral ovarian torsions.

Here, we present the case of a transgendered patient with bilateral ovarian torsions. This case is unique in three ways. First, it is a bilateral ovarian torsion. These are rarely reported, with seven cases listed on PubMed. Of these cases, only three are also simultaneous in presentation [4,7,8]. Second, the patient was a transgender female-to-male, preoperative but on testosterone hormone therapy. This is an emerging patient population and as such may be prone to a generalization bias that is the natural outgrowth of the gestalt discussed above. Finally, and most alarmingly, the patient endorsed only mild-to-moderate pain. This case highlights the importance of having a high clinical suspicion when dealing with unique patient populations.

## Case presentation

A 21-year-old transgender female-to-male patient presented to the emergency department with variable abdominal complaints, including left flank, left lower quadrant, and suprapubic abdominal pain. The pain was described as intermittent, crampy, and generalized, with occasional sharp intermittent pain in the left lower quadrant. He denied aggravating or alleviating factors. His pain was associated with nausea and vomiting and relieved with bowel movements and leaning forward. He denied having dysuria, urinary frequency or urgency, diarrhea, vaginal discharge, or bleeding. His last menstrual cycle was three months prior to the presentation. The patient was no longer having regular menstrual cycles, had not received gender-confirming surgery, and was receiving testosterone therapy weekly for over one year. He presented to the emergency department twice before within two weeks for similar symptoms but left before further evaluation each time for unknown reasons. The patient had no significant medical history. There was no family history of gynecological problems, including cancers or tumors.

On examination, vital signs were in the normal range (blood pressure 119/83 mmHg, heart rate 80 beats/minute, temperature 36.9°C, respiratory rate 18 breaths/minute, and body mass index 22.67 kg/m^2^). He appeared comfortable but had tenderness to the suprapubic area and left pelvic area on physical examination. His abdomen was soft and there were no peritoneal signs. Speculum and bimanual examination revealed cervical motion tenderness and bilateral adnexal tenderness; there was no blood in the vaginal vault.

Investigations

**Figure 1:**
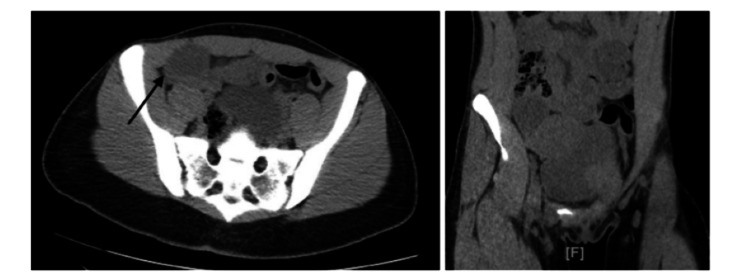
Computed tomography imaging showing the right and left lower quadrant ovarian cysts.

Computed tomography (CT) of the abdomen and pelvis without contrast, as seen in Figure 1, revealed a 3.8 × 2.8 cm cystic structure in the right lower quadrant, a left adnexal dermoid cyst with soft-tissue components measuring 4 × 3 × 3.5 cm, and an adjacent cystic component measuring 7 × 9.3 × 0.1 cm.

His serum and urine pregnancy tests were negative. His complete blood counts and serum biochemistry were within normal limits.

Gynecology was consulted, and a limited transvaginal bedside pelvic ultrasound revealed a left ovarian cyst measuring 8.4 × 7.24 cm with absent Doppler flow, and a right ovary measuring 3.67 × 3.32 cm. There was a concern for ovarian torsion and he was taken to the operating room for exploratory laparotomy.

Treatment

He underwent an exploratory laparotomy with the gynecology team. A large clear mass with calcifications was noted to be dextrorotated with the left ovary, pedicle, and fallopian tube by 360 degrees; the mass and ovary measured 14 × 12 cm together. The ovary was levorotated, and a left salpingo-oophorectomy was performed. The right ovary was also noted to be dextrorotated on the pedicle about 180 degrees. The right ovary was noted to be dextrorotated about 180 degrees on its pedicle and included a hemorrhagic cyst, which measured 8 × 5 cm together. The right fallopian tube and ovary were levorotated and a right cystectomy was performed. There were no intraoperative complications. The patient was discharged on the third postoperative day. He had an uneventful postoperative recovery period. The pathology report later revealed a left mature teratoma measuring 11 cm and a right hemorrhagic corpus luteal cyst.

## Discussion

Ovarian torsion is a gynecological emergency that requires evaluation and treatment within six hours to optimize reproductive organ salvage. Cases of bilateral ovarian torsion are exceedingly rare, making this case unique in and of itself. The few cases that have been documented have typically occurred asynchronously. To the authors’ knowledge, there has never been a documented case of bilateral ovarian torsion in the transgender population.

Although the rarity of this case is certainly worth discussing, the authors are more concerned with the uniqueness of this patient’s presentation. In most cases of complete torsion, presentations will trigger physician gestalt due to the severity of patient distress. Alarmingly, in this case, the patient described his pain as mild, and he was not in distress. The reasons for this cannot be ascertained in a single case report, but highlight the importance of maintaining a high index of suspicion, particularly in emerging patient populations. Despite this, it is worth considering these potential reasons as they can help providers maintain a high index of suspicion. The first reason is that the ovarian torsion pain may improve late in the course of the disease, well past the window of ovarian salvage. However, even though our patient had multiple emergency department visits and against medical advice (AMA) departures, he was able to have one ovary saved. This indicates a likely intermittent torsion prior to arrival.

It is important to note the population identified in this case. The patient is a transgender male who visited multiple emergency departments and had a delayed diagnosis possibly due to several reasons. These reasons may include the patient leaving AMA before further evaluation could be performed; the perception of the patient’s pain/clinical picture not matching the “expected” presentation of ovarian torsion; the patient’s unwillingness to endorse true pain level for fear of being gendered [9,10]; and, lastly, the inexperience with this condition in such populations. Cases like this can perhaps be seen as a challenge to all generalizations, as every patient is unique. This theme has been touched on before when generalizing the results of medical trials outside of the trial ethnic group [10].

There are a number of cases describing the occurrence of asynchronous bilateral ovarian torsion in females; however, there are close to no reported cases regarding the possible effects of testosterone on the maturity of ovarian follicles in transgender male populations and in populations without existing PCOD [2,3,5,6]. In animal trials, testosterone has been observed to cause proliferative ovarian effects that mimic the pathophysiology of PCOD in humans which in extension and in relation to our case discussion may implicate testosterone being a possible contributing factor [11,12]. A study by Caaneen et al. showed no significant differences in the morphology and size of ovarian follicles in adult females undergoing at least one year of testosterone treatment as compared to the control population [13]. With contrary information regarding the effect of testosterone on ovarian cysts, more studies are needed to elucidate the true role of testosterone in this pathology. Additionally, there are no studies centered on transgender male populations, or other populations like body-builders and competitive athletes, more likely to use exogenous testosterone.

## Conclusions

Ovarian torsion is a rare and diagnostically challenging diagnosis in the emergency department even when the patient presents with the “classic” symptomatology. This case report highlighted an unusual case in that it was a transgendered male with bilateral torsion.

Physician gestalt is a critical component of emergency care and is proven to be quite accurate in numerous instances. However, when wrong, gestalt can have serious consequences for patients. As such, in emergency medicine, if we are right, it is a gestalt. If we are wrong, it is a bias. If we are right, it is a heuristic. If we are wrong, it is a shortcut. These retrospective truths are often indiscernible from a prospective view. A better understanding of our pattern recognition is crucial when approaching emerging patient populations. It is possible that prior patient experiences are not generalizable to these emerging populations, as demonstrated in this case. This raises the question of how these patients can best be managed in a busy emergency department with limited resources. We advise a high index of suspicion until more data, both objective and subjective, are known.
